# Patient Perception of Prolapse Condition Questionnaire: A Validated Patient-Reported Outcome Measure

**DOI:** 10.1007/s00192-024-05957-3

**Published:** 2024-10-30

**Authors:** Gans Thiagamoorthy, Rayan Mohamed-Ahmed, Maria Vella, Linda Cardozo, Ilias Giarenis, Martino Zacche, Richard Flint, Sushma Srikrishna, Dudley Robinson

**Affiliations:** 1 Department of Urogynaecology, St Peter’s Hospital, Chertsey, KT16 0PZ UK; 2 Department of Urogynaecology, Golden Jubilee Wing, King’s College Hospital, London, SE5 9RS UK; 3 Department of Urogynaecology, West Suffolk Hospital, Suffolk, IP33 2QZ UK; 4 Department of Urogynaecology, Norfolk and Norwich University Hospital, Norwich, NR4 7UY UK; 5 Department of Urogynaecology, James Pages University Hospital, Great Yarmouth, NR31 6LA UK; 6 Department of Gynaecology, Chelsea and Westminster Hospital, London, SW10 9NH UK

**Keywords:** Prolapse, PROMs, Quality-of-life, Validation

## Abstract

**Introduction and Hypothesis:**

Identifying patient-reported outcome measures allows management of urogenital prolapse to be tailored to reflect symptom bother and expectations of treatment. We devised a new single-item questionnaire, the Patient Perception of Prolapse Condition (PPPC), based on the Patient Perception of Bladder Condition (PPBC). The aim was to evaluate the criterion validity, test/re-test reliability and responsiveness of the PPPC.

**Methods:**

Women attending a tertiary urogynaecology clinic were recruited. At visit 1, patients completed the Prolapse Quality of Life (P-QOL) and PPPC questionnaires, and underwent a Pelvic Organ Prolapse Quantification (POP-Q) examination. This allowed assessment of criterion validity using Spearman’s rank correlation (rho) of the PPPC against validated subjective and objective outcomes. At visit 2, within the next 6 weeks, PPPC was repeated to assess test/re-test reliability using Cronbach's alpha (α). In those undergoing pelvic floor surgery, responsiveness of the PPPC was assessed at visit 3 by correlating PPPC and P-QOL scores 6 weeks post-operatively.

**Results:**

A total of 178 patients attended visit 1, 60 attended visit 2 and 58 attended visit 3. At visit 1, there were moderate correlations between the PPPC and both objective (POP-Q: rho = 0.385, *p* < 0.01, CI 0.192–0.549) and subjective (P-QOL: rho = 0.635, *p* < 0.01, CI 0.493–0.744) measures confirming criterion validity. Test/re-test reliability was high (α = 0.89). Correlation with post-operative PPPC and P-QOL confirmed moderate responsiveness (rho = 0.54, *p* < 0.01).

**Conclusion:**

The PPPC, a novel single-item patient-reported measure of prolapse condition, demonstrated good criterion validity, test/re-test reliability and responsiveness. These findings support the use of the PPPC as a global assessment of prolapse condition.

## Introduction

Patient-reported outcome measures (PROMs) have been recommended by a multitude of women’s health organisations, including the Royal College of Obstetricians and Gynaecologists and the International Continence Society [1]. They are completed by the patient so should ideally represent the patient’s own unique personal perception without clinician bias. Clinicians' assessments of patients' outcomes have at times underestimated the severity of bother perceived by patients and have overestimated the effect of surgical treatment [2, 3]. The latest International Consultation on Incontinence stated that PROMs form “the basis of the patient-centred health care system and represent the most important clinical review of symptom impact and treatment benefit from a patient perspective” [1].

There are currently many different PROMs for assessing the impact of prolapse on a woman’s quality of life. They tend to be long questionnaires, such as the Pelvic Floor Impact Questionnaire (PFIQ) [4] or Pelvic Organ Prolapse Distress Inventory (POPDI), with 93 and 46 question items respectively. Shortened versions of both these questionnaires, the PFIQ-7 and POPDI-6, are now available, containing 7 and 6 items respectively. Health literacy is a concern and employees of the National Health Service (NHS) are advised to pitch the level of written information to the reading age of under 9 years old. At this level only 85% of the adult English reading population can follow it [5]. Lengthy questionnaires may be difficult for the patient to complete owing to literacy issues, the age of our patient population and general willingness to engage with health care.

Previous research has found that “single-item global questions” are practical owing to their brevity, ease of use and ease of interpretation [6]. They have been undervalued because of concerns that there may be a lack of internal consistency, reliability and carefully evaluated measurement properties [7]. However, it has been shown that when completing single-item global questionnaires, the users can consider all aspects of the problem that they are contemplating and provide a true reflective answer [7].

During a literature review to assess the different questionnaires available, the benefit of the Patient Perception of Bladder Condition (PPBC) was evaluated [7]. The PPBC is a single-item global measure that was used in overactive bladder drug trials. It was originally used in the development of duloxetine for SUI. It was translated into 22 different languages and subsequently validated, confirming internal consistency, reliability and appropriate measurement properties. The outcome of interest with PPBC was bladder condition, different to the outcome we sought to assess, prolapse condition. As the ideal PROM should be specific to the condition being assessed the PPBC was not suitable for our needs. As there were no other single-item global questionnaires for prolapse that could be used at any stage in the patient’s journey, a new PROM, specific to prolapse was required.

The aim of this study was to devise and validate a single-item global questionnaire to assess the effect of prolapse on our patients’ quality of life.

## Materials and Methods

### Devising a New PROM

**Fig. 1:**
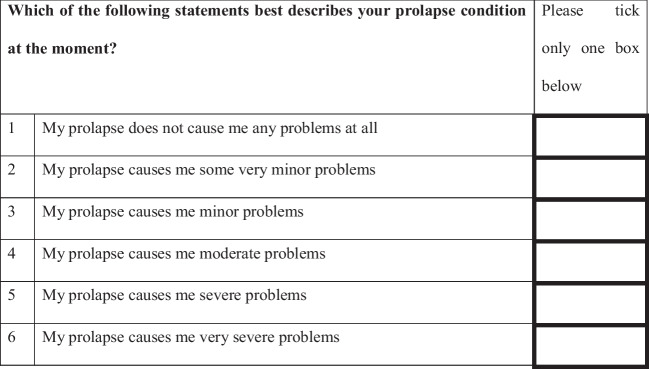
Patient Perception of Prolapse Condition questionnaire

We devised a new questionnaire, the Patient Perception of Prolapse Condition (PPPC), based on adaptation of the PPBC. The PPPC is a single-question, six-point global scale in which patients rate their current impression of their prolapse (Fig. 1). Patients are asked to describe their prolapse on a six-point scale ranging from 1, corresponding to “does not cause me any problems at all” to 6, which corresponds to “causes me severe problems”.

### Content Validity

To ensure content validity the pilot PROM was developed within the urogynaecology multidisciplinary environment of a tertiary referral centre. This was assessed by a lay person in order to ensure ease of reading and understandability. The pilot PROM was then assessed via interviews with 20 patients who had agreed to provide feedback; these patients were recruited from the urogynaecology outpatient clinic. The questions were then modified following the feedback provided, which mostly related to ease of understanding, and a final draft created. The questionnaires were ratified once more by the urogynaecology multidisciplinary team prior to distribution.

### Determining the Mode of Administration

The mode of administration of the PROM is important, as changing the mode may lead to a variation in the responses. It has been suggested that intimate questions might better completed when self-administered rather than interviewer administered as the patient may not feel able to express her true thoughts out loud to the interviewer [1]. Also, as our PROM is to assess the bother caused by prolapse at any stage in the patient’s care, i.e. before or after interventions, the patient may feel obliged to show appreciation for the efforts made by the clinician to treat her and not provide a true response. There is also the risk of the interviewer leading the patient by the wording of their questions during completion of the PROM, or even through unintended intonation of their voice. As such, it was decided that the PROM would be self-administered.

The mode of self-administration was also taken into account. A questionnaire that is completed with a pen and paper may theoretically elicit different responses when the same questionnaire is administered via a screen and mouse or touchscreen device [8]. The initial thought was to create an electronic PROM; however, assessing the demographics of our patient population and our decision that it would best be self-administered, we deemed that an electronic PROM would exclude a section of our population. As such, the questionnaire would be conducted via pen and paper.

### Formal Validation

As this was a novel questionnaire, the validity of the original questionnaire (PPBC) did not apply. To ensure that the information gained via this PROM was clinically useful, the PPPC needed to be validated in women with POP.

The International Consultation on Incontinence (ICI) states that the development of a PROM requires a multistep, structured process, with studies that demonstrate the measure's validity, reliability and responsiveness [1]. To validate the PPPC questionnaire, we needed to evaluate criterion validity, test/re-test reliability and its responsiveness. The psychometric validation of PROMs is described within the literature [9]. The ICI was utilised as a manual to conduct the validation of the PPPC and the definitions used are from those guidelines.

Validity refers to the ability of an instrument to measure what it is intended to measure.

- Criterion validity reflects the correlation between the new questionnaire, PPPC and an accepted reference, or gold standard [1]. In this regard, we decided to assess the criterion validity looking at both subjective and objective measures. For the subjective measure we utilised the Prolapse Quality of Life (P-QOL) and for the objective criterion validity we utilised detailed examination of prolapse by the Pelvic Organ Prolapse Quantification (POP-Q) ordinal staging system. When criterion validity is to be established using an existing measure, the correlation, assessed using Spearman’s rank correlation should be 0.40 to 0.70 [10]. It has been stated that correlations approaching 1.0 indicate that the new questionnaire is too similar to the gold standard and as such is not required.
- Reliability or test–retest reliability is defined as the ability of a PROM to produce similar results when undertaken under the same circumstances repeatedly. To assess test–retest reliability, the same patient completes the questionnaire more than once, at baseline and again after a period of time during which the impact of the symptoms is unlikely to change (e.g. a few days or weeks) [11]. Cronbach’s alpha is used to demonstrate reproducibility and α between 0.7 and 0.9 is considered “good”, whereas α greater than 0.9 is considered “excellent” [1].
- Responsiveness indicates whether the measure can detect change (improvement or deterioration) in a patient's condition. To assess this the patients are utilised as their own controls and the PROM conducted once more after undergoing an intervention.

To undertake validation of the PPPC a prospective case-controlled study was conducted. Women were recruited from a tertiary urogynaecology clinic over an 8-month period. All women over the age of 18 years, literate in English, presenting with urogynaecological symptoms and able to provide informed written consent were invited to participate. There were no exclusion criteria.

At visit 1, prior to seeing a clinician for history and assessment, the patients referred for prolapse concerns completed the P-QOL and PPPC questionnaires. During the assessment they underwent a POP-Q examination in both the lying and the standing position at maximum Valsalva. The most severe extent of their prolapse was recorded. This enabled assessment of criterion validity comparing PPPC with P-QOL for subjective assessment, whereas objective assessment was conducted comparing the PPPC with the POP-Q. As the scores did not follow a normal distribution, criterion validity was assessed using Spearman’s rank correlation (rho) of the PPPC against the validated subjective and objective outcomes.

At visit 2, between the next 4 to 8 weeks, the PPPC was repeated to assess test/re-test reliability using Cronbach's-alpha (α).

Some of the women went on to undergo surgery for POP. In those undergoing pelvic floor surgery, the responsiveness of the PPPC was assessed at visit 3 by correlating PPPC and P-QOL scores 6 weeks post-operatively.

Statistical analysis was carried out using MedCalc Statistical Software version 12.7.8.

## Results

In total, 178 patients were recruited. Our population had a median age of 56 years (range 27–89 years), median BMI 26.4 (19.5–48.7) and median parity 2 (0–6). The median Ordinal stage POP-Q was 2 (range 0–4).

All 178 women attended visit 1, 60 attended visit 2 and 58 attended visit 3.

**Fig. 2:**
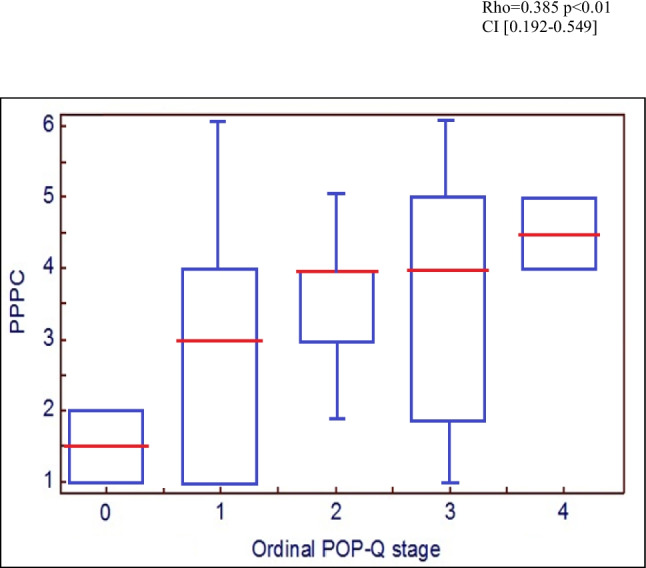
Criterion validity: correlation of Patient Perception of Prolapse Condition (PPPC) and Pelvic Organ Prolapse Quantification (POP-Q; objective)

**Fig. 3:**
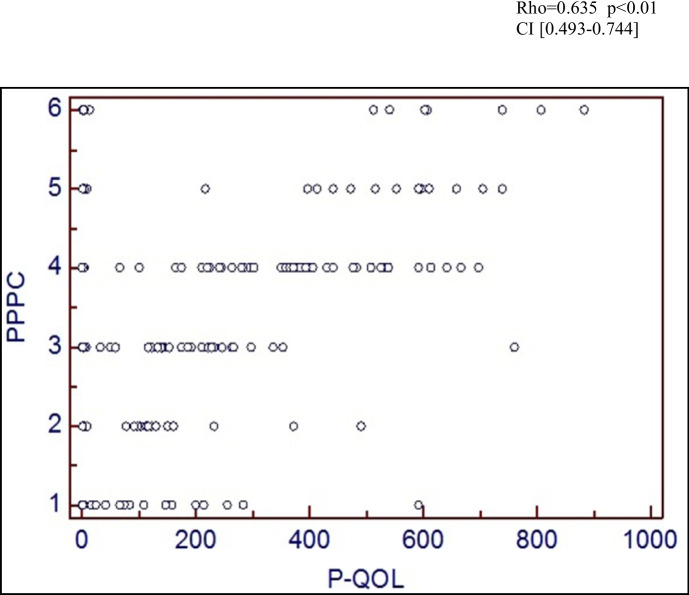
Scatter graph to illustrate criterion validity: correlation of Patient Perception of Prolapse Condition (PPPC) and Pelvic Organ Prolapse Quantification (P-QOL; subjective)

**Fig. 4:**
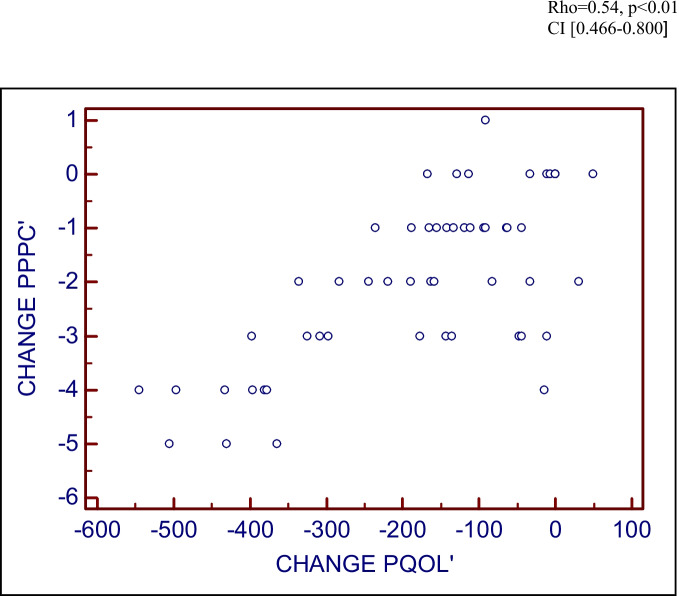
Responsiveness: assessing correlation between change in pre- and post-operative Patient Perception of Prolapse Condition (PPPC) and pre- and post-operative Pelvic Organ Prolapse Quantification (P-QOL) scores

At visit 1, there were excellent correlations between the PPPC and both objective (POP-Q: rho = 0.385, *p* = 0.01, CI 0.192–0.549) and subjective (P-QOL: rho = 0.635, *p* < 0.01, CI 0.493–0.744) measures, confirming criterion validity (Figs. 2 and 3). A box and whisker chart is used to demonstrate objective correlation rather than a scatter graph as the ordinal nature of the POP-Q resulted in the plots overlaying, rendering the scatter graph unillustrative. Assessing correlation between the PPPC at two different points in time before any intervention confirmed test/re-test reliability. Correlation was good, Cronbach’s α = 0.89 (0.7 ≤ α < 0.9 good, α > 0.9 excellent) confirming test/re-test reliability of The PPPC. The PPPC was highly responsive to proven post-operative improvements. This responsiveness was demonstrated in Fig. 4, in which there was moderate correlation between post-operative PPPC and P-QOL (rho = 0.54, *p* < 0.01, CI 0.466–0.800).

## Discussion

With recent controversy surrounding urogynaecological surgery, especially allegations of unnecessary surgery, the use of mesh and subsequent litigation, it is essential that clinicians are absolutely sure of what they should offer patients. To do this, it is vital that clinicians return to first principles and clearly assess patients in an unbiased and non-leading manner so that they understand what exactly concerns the patient and how severely it bothers her. PROMs are tools that allow for exactly that, as they are completed by the patient and represent the patient’s own unique personal perception.

The existing PROMs for use in women with POP provide a detailed range of important information on the effect of prolapse on various aspects of their life, but can be difficult for them to complete, cumbersome for the clinician to process and not ideal in our short NHS clinic appointment time, especially now that the amount of administration required within the appointment time is ever increasing. Global, single-question PROMs, such as the Patient Global Impression of Improvement (PGI-I) after treatment, are practical and have been shown to encompass consideration of all symptoms [12]. Owing to their brevity they are ideal in the modern NHS. Such brevity may increase clinician’s willingness to request their patients to kindly take a minute or so to answer the question. The brevity may also increase the chances of the patient completing it. The patient completing the PROM themselves also reduces the chance of bias from surgeons, both pre- and post-operatively. This can then be used to audit the success rate of surgeons and their teams and, if combined onto a national platform, such as the British Society of Urogynaecology database, it will assist in auditing a particular clinician’s or hospital team’s outcomes against the national average.

The development of a PROM is a rigorous, scientific process to provide confidence that the PROM is measuring what it is intended to measure, that it does this reliably and is appropriate for use in the patient or population group under investigation. The final instrument must demonstrate validity, reliability and responsiveness in the intended target population. The PPPC, our global assessment index of prolapse severity, has performed well and demonstrated validity when compared with POP-Q and P-QOL; confirmed test/re-test reliability when assessed at two different time points; and responsiveness with good correlation between the post-operative PPPC and P-QOL. The PPPC can as such be used at different time points in the pathway of a patient who is suffering with POP.

The PPPC was modelled on the PPBC, which was devised and has been used in a number of overactive bladder medication trials. The authors of the PPBC stated that the simplicity and usefulness of the PPBC were recognised, but that limitations of single-item global measures existed [7]. The weakness of a single-item global index is that it is unable to elicit the different aspects of the woman’s life that are affected by prolapse in as much detail as the longer PROMs are capable of doing. Highlighting each individual problem can assist history taking and also leave the woman more content after the consultation, as the clinician can seek to resolve each aspect. Multiple-item PROM may also have a role in assessing changes post-surgery, where multiple POP compartments have been causing symptoms. For example, a young woman with anterior and uterine prolapse but mild posterior prolapse may have undergone a vaginal hysterectomy and anterior repair, without any surgery to the posterior wall. The multiple component questionnaire may highlight better than a single-item questionnaire if the patient did or did not still have bothersome bowel symptoms, which may help the clinician to consider an interval posterior repair. This information could be obtained during the consultation as well. The authors of the PPBC also highlighted an important point that use of specific PROMs do not need to be mutually exclusive. Use of the PPPC as a single-item PROM may well be utilised with a multi-item PROM if the individual scenario required it, and both may complement one another.

Limitations of our study include the non-digital nature of the PROM, which we felt was more user friendly in our cohort. Re-validation for the PROM would be required if an electronic version were to be reintroduced. There was a high attrition rate between visits 1 and 2, which means that the reliability of the tool was demonstrated on a smaller population. Although we feel that the PPPC is an important addition to the pre-existing PROMs available in urogynaecology, it is also important to consider the importance of using a limited number of questionnaires across research purposes, in order to standardise assessment and draw conclusions from wide ranges of research [13].

## Conclusion

The ideal PROM should be specific to the condition being assessed. To our knowledge, the PPPC is the first single-item global index PROM that can be used to assess the effect of POP on quality of life at any stage of the patient’s journey. This novel questionnaire has the minimum number of items, a single question, where the patient chooses just one answer that best suits her current situation from six options. This single-item patient-reported measure of prolapse condition has been demonstrated to have moderate criterion validity when compared with objective (POP-Q) and subjective (P-QOL); very good test/re-test reliability when assessed at different points in time; and responsiveness to change via improvements between scores before and after surgery. These findings support the use of the PPPC as a global tool for the assessment of prolapse condition.

## References

[1] Abrams P, Cardozo L, Wagg A, Wein A. Incontinence. 7th ed. Bristol: International Continence Society; 2023. p. 437–86.

[2] Rodríguez LV, Blander DS, Dorey F, Raz S, Zimmern P. Discrepancy in patient and physician perception of patient’s quality of life related to urinary symptoms. Urology. 2003;62(1):49–53. 12837421 10.1016/s0090-4295(03)00144-4

[3] Black N, Griffiths J, Pope C, Bowling A, Abel P. Impact of surgery for stress incontinence on morbidity: cohort study. BMJ. 1997;315(7121):1493–8. 9420489 10.1136/bmj.315.7121.1493 PMC2127933

[4] Barber MD, Kuchibhatla MN, Pieper CF, Bump RC. Psychometric evaluation of 2 comprehensive condition-specific quality of life instruments for women with pelvic floor disorders. Am J Obstet Gynecol. 2001;185(6):1388–95. 11744914 10.1067/mob.2001.118659

[5] DfBI Skills. The 2011 Skills for Life Survey: a survey of literacy, numeracy and ICT levels in England. 2012.

[6] Sloan JA, Aaronson N, Cappelleri JC, Fairclough DL, Varricchio C. Assessing the clinical significance of single items relative to summated scores. Mayo Clin Proc. 2002;77(5):479–87. 12004998

[7] Coyne KS, Matza LS, Kopp Z, Abrams P. The validation of the patient perception of bladder condition (PPBC): a single-item global measure for patients with overactive bladder. Eur Urol. 2006;49(6):1079–86. 16460875 10.1016/j.eururo.2006.01.007

[8] Coons SJ, Gwaltney CJ, Hays RD, Lundy JJ, Sloan JA, Revicki DA, et al. Recommendations on evidence needed to support measurement equivalence between electronic and paper-based patient-reported outcome (PRO) measures: ISPOR ePRO Good Research Practices Task Force report. Value Health. 2009;12(4):419–29. 19900250 10.1111/j.1524-4733.2008.00470.x

[9] Christensen KB, Comins JD, Krogsgaard MR, Brodersen J, Jensen J, Hansen CF, et al. Psychometric validation of PROM instruments. Scand J Med Sci Sports. 2021;31(6):1225–38. 33341986 10.1111/sms.13908

[10] Mukaka MM. Statistics corner: a guide to appropriate use of correlation coefficient in medical research. Malawi Med J. 2012;24(3):69–71. 23638278 PMC3576830

[11] Revicki DA, Osoba D, Fairclough D, Barofsky I, Berzon R, Leidy NK, et al. Recommendations on health-related quality of life research to support labeling and promotional claims in the United States. Qual Life Res. 2000;9(8):887–900. 11284208 10.1023/a:1008996223999

[12] Guy W. ECDEU Assessment Manual for Psychopharmacology. Rockville, MD: US Department of Health, Education, and Welfare Public Health Service Alcohol, Drug Abuse, and Mental Health Administration, 1976.

[13] Grigoriadis T, Athanasiou S, Rizk D. Female pelvic floor dysfunction questionnaires: the modern Tower of Babel? Int Urogynecol J. 2020;31(6):1059–61. 31989203 10.1007/s00192-019-04220-4

